# Increasing the bioflocculant production and identifying the effect of overexpressing *epsB* on the synthesis of polysaccharide and γ-PGA in *Bacillus licheniformis*

**DOI:** 10.1186/s12934-017-0775-9

**Published:** 2017-09-26

**Authors:** Peize Liu, Zhen Chen, Lijie Yang, Qingbiao Li, Ning He

**Affiliations:** 1 Department of Chemical and Biochemical Engineering, College of Chemistry and Chemical Engineering, Xiamen University, Xiamen, 361005 People’s Republic of China; 2 The Key Lab for Synthetic Biotechnology of Xiamen City, Xiamen University, Xiamen, 361005 People’s Republic of China

**Keywords:** *Bacillus licheniformis*, Polysaccharide, *epsB*, γ-PGA, Gene overexpression

## Abstract

**Background:**

Polysaccharides and poly-γ-glutamic acid (γ-PGA) are biomacromolecules that have been reported as bioflocculants, and they exhibit high flocculating activity in many industrial applications. *Bacillus licheniformis* CGMCC 2876 can produce polysaccharide and γ-PGA bioflocculants under different culture conditions. Several key genes are involved in the metabolic pathway of polysaccharides in *B. licheniformis*, but the impacts of the regulation of these genes on the production of polysaccharide bioflocculants have not been illustrated completely. To increase the bioflocculant production and identify the correlation between the synthesis of polysaccharides and γ-PGA in *B. licheniformis*, a few key genes were investigated to explore their influence on the synthesis of the bioflocculants.

**Results:**

Overexpressing *epsB* from the *eps* gene cluster not only improved the bioflocculant crude yield by 13.98% but also enhanced the flocculating activity by 117.92%. The composition of the bioflocculant from the *epsB* recombinant strain was 28.95% total sugar, 3.464% protein and 44.03% γ-PGA, while in the original strain, these components represented 53.67%, 3.246% and 34.13%, respectively. In combination with an analysis of the transcriptional levels of several key genes involved in γ-PGA synthesis in *B. licheniformis*, we inferred that *epsB* played a key role in the synthesis of both polysaccharide and γ-PGA. The bioflocculant production of the *epsB* recombinant strain was further evaluated during batch fermentation in a 2 L fermenter; the flocculating activity reached 9612.75 U/mL, and the bioflocculant yield reached 10.26 g/L after 72 h, representing increases of 224% and 36.62%, respectively, compared with the original strain. Moreover, we found that the tandem expression of phosphoglucomutase (*pgcA*) and UTP-glucose-1-phosphate uridylyltransferase (*gtaB1*) could enhance the crude yield of the bioflocculant by 20.77% and that the overexpression of *epsA* could enhance the bioflocculant yield by 23.70% compared with the original strain.

**Conclusions:**

This study provides a new method to greatly increase the bioflocculant production in *B. licheniformis*, and it demonstrates the correlation between the biosynthesis of polysaccharide and γ-PGA during EPS fermentation by regulating the expression of EpsB.

**Electronic supplementary material:**

The online version of this article (doi:10.1186/s12934-017-0775-9) contains supplementary material, which is available to authorized users.

## Background

Polysaccharides are biomacromolecules that are produced by a wide variety of microorganisms, and they have recently attracted increasing attention because of their unique characteristics, such as their biodegradability, nontoxicity and negligible secondary pollution [1]. Polysaccharides have been reported as bioflocculants for many potential applications, such as for wastewater treatment and drinking water purification and in the food additive and fermentation industries [2, 3]. Although polysaccharide bioflocculants have many advantages, the low yield and high cost of their production as flocculants have largely limited their industrial applications [4].

Poly-γ-glutamic acid (γ-PGA) is a naturally occurring macromolecular polyamide that consists of d/l-glutamate monomers [5, 6]. γ-PGA exhibits many favourable features such as biodegradability, water solubility, edibility and nontoxicity to humans and the environment [7]. Like polysaccharides, γ-PGA can also be used as a bioflocculant in many applications, such as food, cosmetics and agriculture [5].

*Bacillus licheniformis* is a gram-positive, spore-forming soil bacterium that can secrete many kinds of biopolymers that can serve as antibiotics, biochemicals and consumer products, among other purposes [8, 9]. In our previous research, a bioflocculant-producing bacterium was isolated and identified as *B. licheniformis* [China General Microbiological Culture Collection Centre (CGMCC) 2876] [10], and it could produce polysaccharide and γ-PGA under different culture conditions. When using glucose as the sole carbon source, *B. licheniformis* mainly produces extracellular polysaccharides [11], whereas when using trisodium citrate and glycerol as the carbon sources, this strain mainly secretes γ-PGA [12]. Both polysaccharides and γ-PGA have shown high flocculating activities in industrial applications, so the ability to increase the flocculating activities of bioflocculants produced by *B. licheniformis* has huge commercial potential [13].

In this study, we mainly addressed bioflocculants that use glucose as the sole carbon source, and we used genetic engineering as a means to improve the flocculating activity and crude yield of the bioflocculants. In our previous research, we suggested the hypothetical metabolic pathways of polysaccharides in *B. licheniformis* and speculated on the key genes involved in polysaccharide synthesis; these genes are distributed in different locations in the genome and are responsible for the conversion of glucose into the subunits of polysaccharides [11]. Among the genes, *crr* encodes the protein component of glucose-specific phosphotransferase, whose function is to catalyse d-glucose to glucose-6-phosphate; *pgcA* encodes phosphoglucomutase, which is involved in converting glucose-6-phosphate to glucose-1-phosphate; then, glucose-1P is converted into UDP-glucose by UTP-glucose-1-phosphate uridylyltransferase, which is encoded by *gtaB*; further, *epsA* and *epsB* are two gene segments of the *eps* gene cluster, which encodes a series of glycosyltransferases that have different substrate specificities and are all important in the process of synthesizing polysaccharide repeating units. The length of the polysaccharide chain is determined by EpsA and EpsB, and then, the polysaccharides are exported under the action of EpsA [14–16]. These genes are all key genes in the main route for synthesizing EPS on the basis of the polysaccharide metabolic pathways that we have presented. Several studies have reported that regulating the expressions of genes can influence the polysaccharide production by the studied strains. In *Staphylococcus aureus* and *Bacillus subtilis*, the glycolipid production is related to the α-phosphoglucomutase PgcA, the α-glucose-1-phosphate uridyltransferase GtaB [17–19] and the glycosyltransferases YpfP and UgtP [20, 21]. The absence of genes *pgcA, gtaB* or *ypfP* prevents the strains from producing glycolipids [22]. Tang reported a linear relationship between the activity of phosphoglucomutase (PGM) and the production of EPS [23]. In *Lactococcus lactis*, overexpression of the phosphoglucomutase gene and UDP-glucose pyrophosphorylase gene can increase the production of UDP-monosaccharide [24], and in *Streptococcus thermophilus* LY03, the overexpression of these two genes can lead to a nearly 100% increase in the polysaccharide yield [25]. In *Streptococcus pneumoniae*, *galU* (glucose-1-phosphate uridylyltransferase gene) knockout mutants are unable to synthesize a detectable capsule polysaccharide [26]. Further, our previous research also indicated that the overexpression of UDP-glucose pyrophosphorylase gene *gtaB2* in *B. licheniformis* could markedly increase both the yield of the polysaccharide bioflocculant and its flocculating activities [11]. In addition, many researchers have reported the importance of the *eps* gene cluster during polysaccharide synthesis, and it exhibits conspicuous homology across different strains [27, 28]. In *Methylobacillus* sp. strain 12S, EpsA regulates the transcription of methanolan synthesis genes, and the constitutive expression of *epsA* in strain 12S increases EPS production; further, researchers have also found that EpsB appears to act as a glucosyltransferase to catalyse the initial transfer of glucose moieties [29]. Alexander K. W. Elsholz reported that in *Bacillus subtilis*, the EPS synthesis depended on a tyrosine kinase that consisted of a membrane component (EpsA) and a kinase component (EpsB). Moreover, the tyrosine kinase-mediated self-regulation could control the exopolysaccharide production in bacteria [30, 31]. In summary, these genes are all important in the synthesis of EPS, but the impacts of regulating these genes on the production of polysaccharides in *B. licheniformis* have not been determined before. In this report, we focused on the effects of overexpressing single or tandem key genes on the synthesis of polysaccharide bioflocculants, and ultimately, we obtained a high-yield recombinant strain of *B. licheniformis* by overexpressing *epsB*.

## Results and discussion

### The construction of recombinant bacteria

Using electroporation followed by screening on tetracycline-resistant culture plates, several recombinant strains were obtained and called *B. licheniformis*-phY300-*epsB, B. licheniformis*-phY300-*epsA, B. licheniformis*-phY300-*pgcA, B. licheniformis*-phY300-*pgcA*-*gtaB1, B. licheniformis*-phY300-*gtaB1,* and *B. licheniformis*-phY300-*crr*.

**Fig. 1:**
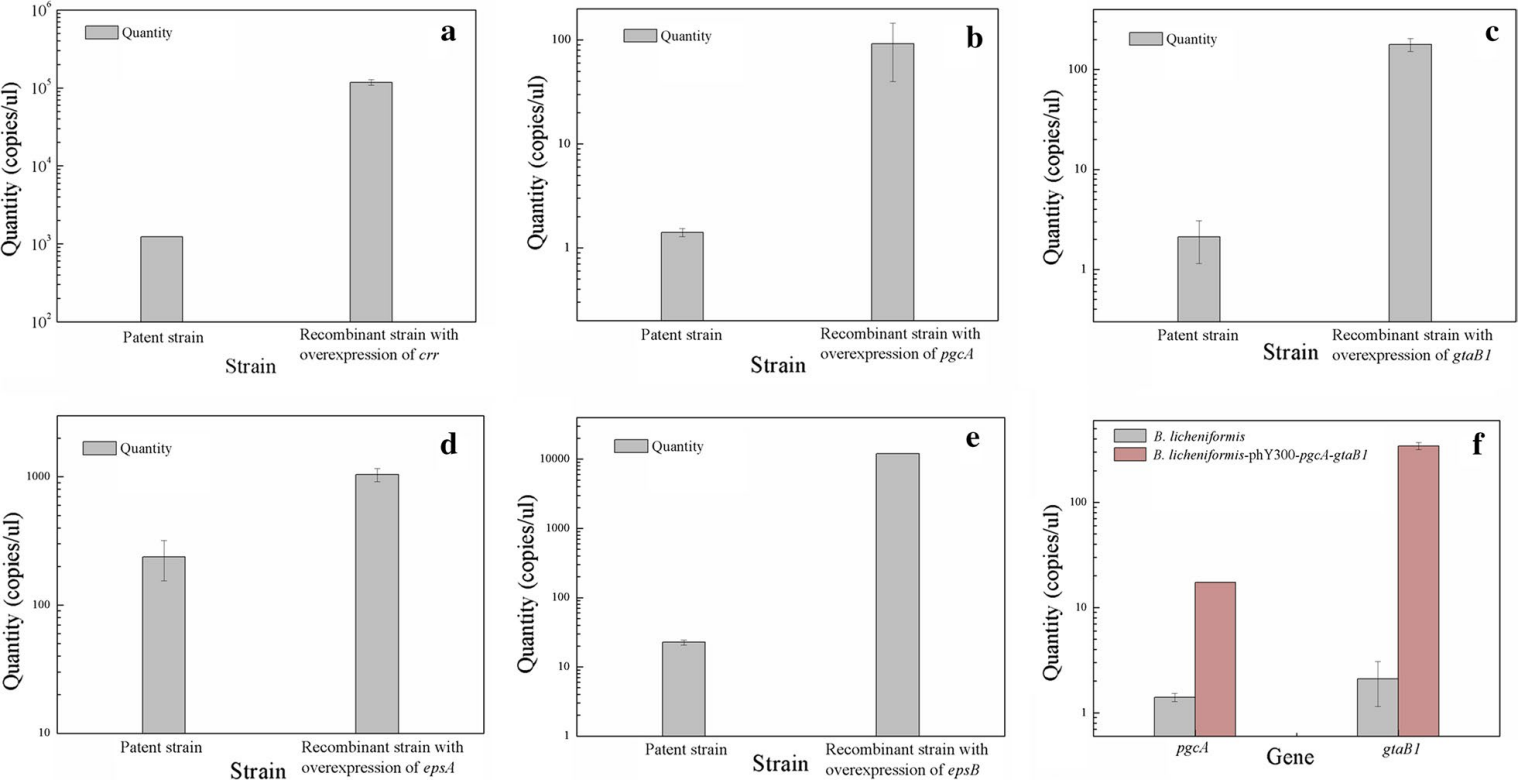
qPCR analysis of key genes in wild-type strain and recombinant strains. **a** *crr*; **b** *pgcA*; **c** *gtaB1*; **d** *epsA*; **e** *epsB*; **f** tandem gene *pgcA*-*gtaB1*

A transcriptional analysis using qRT-PCR showed that the transcriptional levels of all of these key genes in the transformants were markedly increased compared with those of the original strain (Fig. 1).

### Comparison between the original strain and recombinant *B. licheniformis* on the production of the bioflocculants

**Fig. 2:**
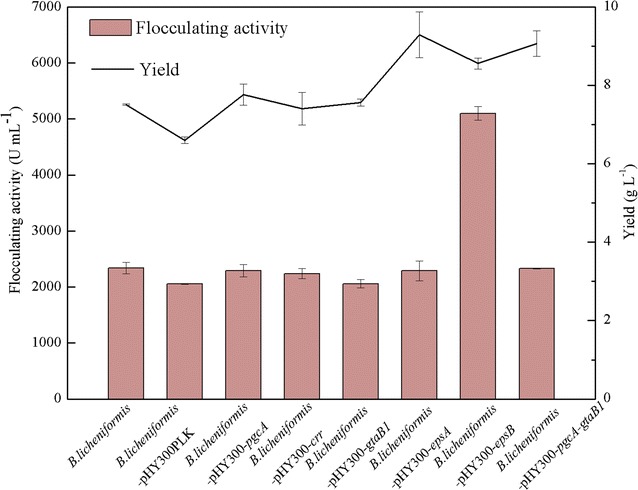
The comparison of original and recombinant *B. licheniformis* on flocculating activity and yield of bioflocculant

As shown in Fig. 2, the crude yield and flocculating activity of the polysaccharide bioflocculant produced by *B. licheniformis* were 7.51 g/L and 2340.52 U/mL, respectively, in the EPS medium for 56 h. The yield and flocculating activity of the bioflocculants produced by the recombinant strains *B. licheniformis*-phY300-*pgcA, B. licheniformis*-phY300-*gtaB1* and *B. licheniformis*-phY300-*crr* were not improved compared with the original strain, demonstrating that solely overexpressing these three genes did not have an obvious impact on the polysaccharide bioflocculant production of *B. licheniformis.* In contrast, many reports have shown that the phosphoglucomutase gene (*pgcA*) and the UDP-glucose pyrophosphorylase gene (*gtaB1*) were key genes of the biosynthetic pathway of polysaccharide in *Bacillus* sp. [24, 25]. We also found that overexpressing these two genes simultaneously increased the bioflocculant crude yield to 9.07 g/L, which was 20.77% greater than that of the original strain. Thus, regulating the expression of genes *pgcA* and *gtaB1* at the same time could promote the production of EPS in *B. licheniformis*. Moreover, the bioflocculant crude yield of *B. licheniformis*-phY300-*epsA* reached 9.29 g/L, which was an increase of 23.70% compared with that of the original strain. Remarkably, overexpressing *epsB* not only improved the bioflocculant crude yield by 13.98% to 8.56 g/L but also enhanced the flocculating activity by 117.92%, to 5100.50 U/mL. Thus, overexpressing *epsB* could dramatically improve the production of bioflocculants in *B. licheniformis.*

### The composition of the bioflocculants produced by *B. licheniformis*-phY300-*epsB*

**Fig. 3:**
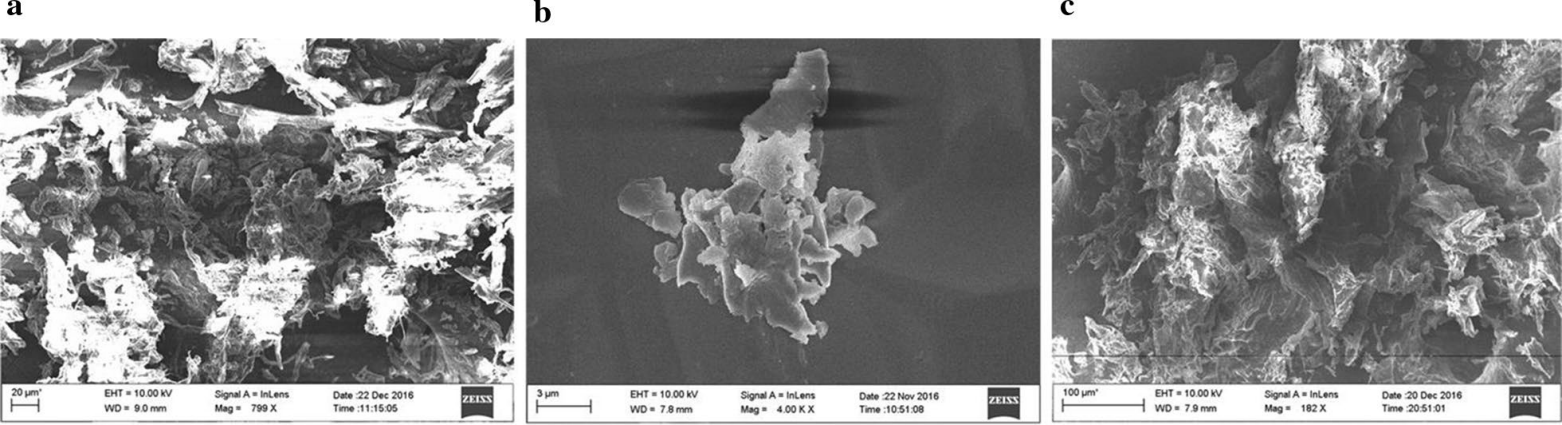
The SEM image of bioflocculant produced by *B. licheniformis* strains. **a** bioflocculant produced by *epsB* recombinant strain. Scale bar = 20 μm; **b** bioflocculant produced by *epsB* recombinant strain. Scale bar = 3 μm; **c** bioflocculant produced by original strain

**Fig. 4:**
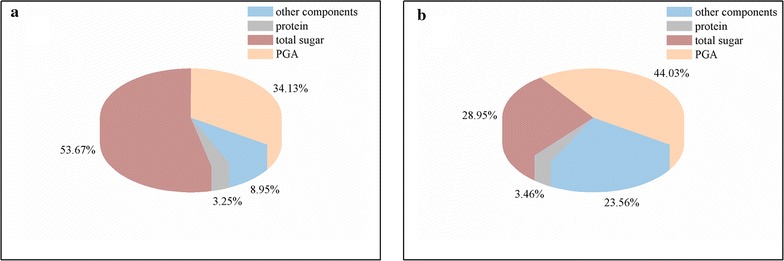
The composition of bioflocculant produced by *B. licheniformis.* **a** wild-type strain; **b** *epsB* recombinant strain

Scanning electron microscope (ZEISS SIGMA, USA) images of the bioflocculants produced by *B. licheniformis*-phY300-*epsB* are shown in Fig. 3. The purified bioflocculant was a white floccus solid after lyophilisation and did not exhibit obvious differences from the bioflocculant produced by the original strain. Meanwhile, the compositions of the bioflocculants produced by the original and *epsB* recombinant strains were markedly different. The total sugar content in the bioflocculant produced by *B. licheniformis*-phY300-*epsB* was 28.95%, the protein content was 3.464%, and the γ-PGA content reached 44.03%. In contrast, in the bioflocculant produced by the original strain, the content of total sugar was 53.67%, the protein content was 3.246%, and the γ-PGA content was 34.13% (Fig. 4). In our previous study, we found that EpsB contained the structural domain of the tyrosine kinase CpsD, which had a negative regulatory role on the formation of capsular polysaccharides in *Streptococcus aureus* [15] according to an analysis of the NCBI Conserved Domains. Therefore, we inferred that *epsB* had a negative regulatory effect on the synthesis of polysaccharides in *B. licheniformis* and thereby decreased the flocculating activity [11]. However, although the most recent results showed that overexpressing *epsB* indeed decreased the polysaccharide content synthesized by *B. licheniformis*, the flocculating activity of the bioflocculant was greatly improved compared with that of the original strain because overexpressing *epsB* produced a marked increase in the γ-PGA content. These results indicated that *epsB* could enhance the synthesis of γ-PGA during EPS fermentation. Moreover, we also found that the content of the other components in the bioflocculant produced by *B. licheniformis*-phY300-*epsB* increased from 8.95 to 23.56%. This result may have been another reason for the improvement in the flocculating activity, but the exact compositions of the active moieties need to be further investigated.

### The effect of overexpressing *epsB* on the biosynthetic pathway of γ-PGA

**Fig. 5:**
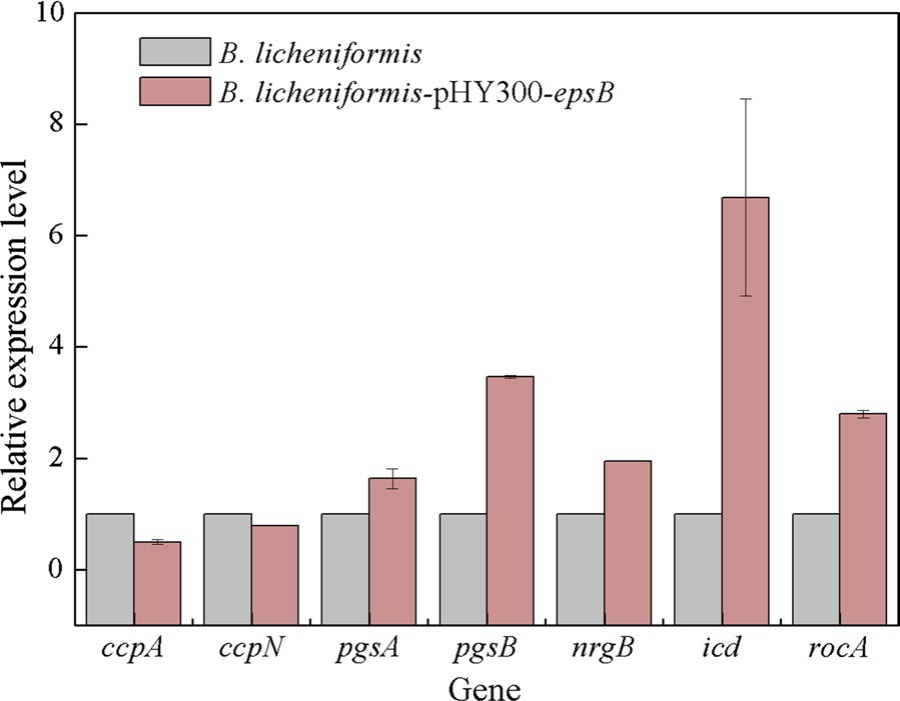
Comparison of the expression level of key genes involved in γ-PGA synthesis between wild-type and *epsB* recombinant strain

**Fig. 6:**
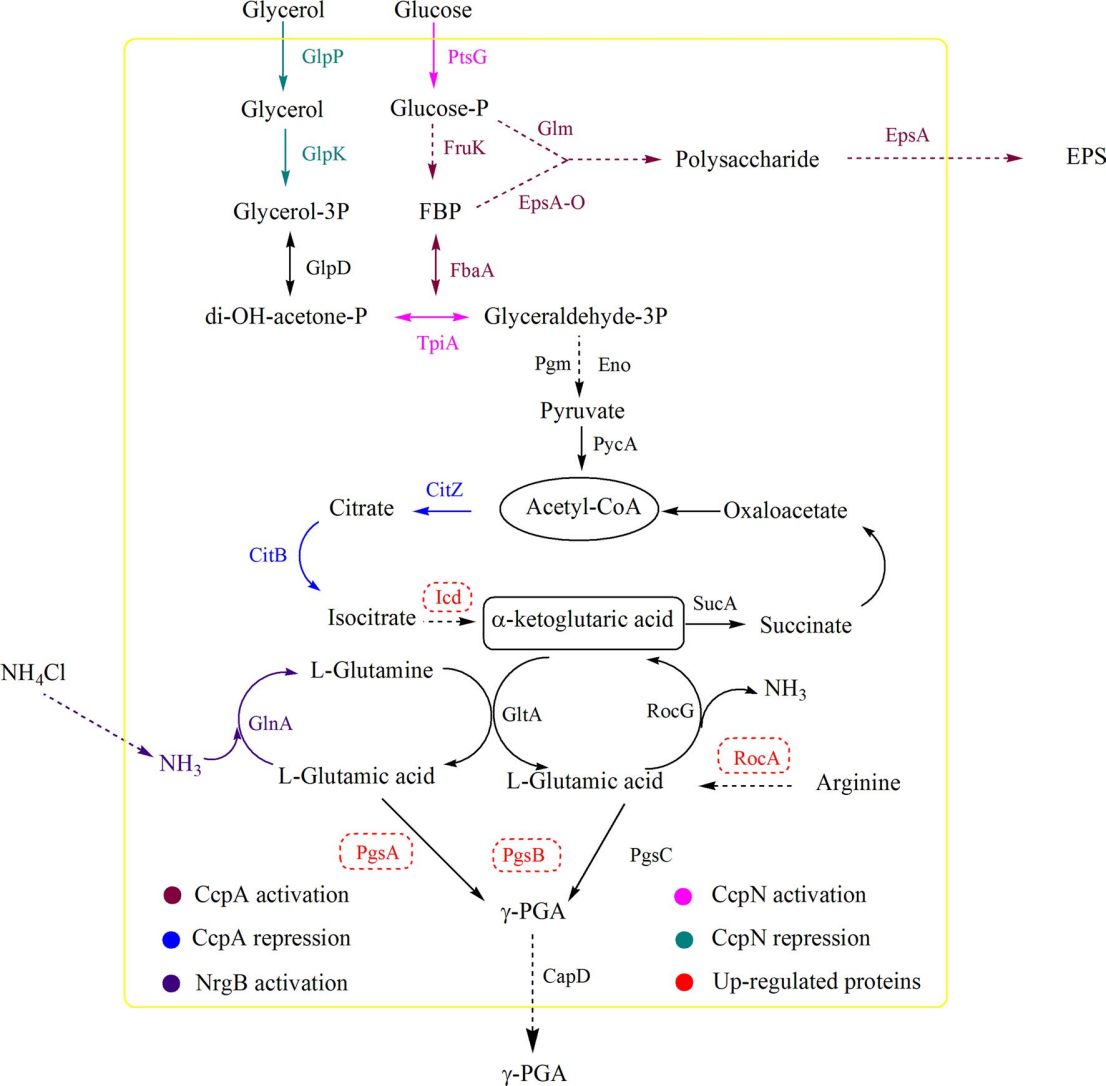
The γ-PGA and polysaccharide synthesis pathways in *B. licheniformis*

To determine the reason for the increase in the γ-PGA content of the polysaccharide bioflocculant when overexpressing *epsB*, the transcriptional levels of a few of the main genes involved in the synthesis of γ-PGA were assayed using quantitative real-time PCR; each sample was performed in triplicate (the related primers are listed in Additional file 1: Table S1). The results are shown in Fig. 5; the gene expression levels of *pgsA*, *pgsB*, *icd*, *rocA* and *nrgB* were increased in the *epsB* recombinant strain compared with the wild-type strain, while the expression levels of genes *ccpA* and *ccpN* were reduced in the *epsB* recombinant strain. Many studies have reported that *pgsA* and *pgsB* are synthetase genes that are involved in the biosynthesis of γ-PGA [32–35]; therefore, we inferred that overexpressing *epsB* could induce the transcription and improve the expression of γ-PGA synthetase in *B. licheniformis*, thereby resulting in an increase in the γ-PGA synthesis. The TCA cycle is an important pathway that is involved in glutamic acid biosynthesis, and isocitrate dehydrogenase, encoded by *icd*, is a rate-limiting enzyme in the process of acetyl-CoA conversion to α-ketoglutaric acid [32]. Moreover, arginine can be transformed to glutamic acid, an important pathway for the biosynthesis of glutamic acid, and the gene *rocA* is involved in this process [32, 36]. Thus, overexpressing *epsB* could also increase the expression of isocitrate dehydrogenase and facilitate the conversion of acetyl-CoA to α-ketoglutaric acid; meanwhile, overexpressing *epsB* could promote the transformation of arginine to glutamic acid. These processes would all eventually increase the γ-PGA synthesis. In contrast, in *B. licheniformis*, the carbon metabolism regulatory proteins CcpA and CcpN and the nitrogen metabolism regulatory protein NrgB played core roles in the transition between the metabolism of the polysaccharide and γ-PGA. CcpA and CcpN could co-enhance glycolysis and suppress the carbon flux in the TCA cycle, consequently slowing the synthesis of γ-PGA; simultaneously, CcpN could cut off the carbon flux from glycerol metabolism and further reduce the γ-PGA production [37]. The synthesis of γ-PGA was also influenced by NrgB, which could transform the major nitrogen metabolic flux between NH_4_^+^ and glutamate, facilitate ammonium utilization and promote glutamine synthesis; these effects are all beneficial for γ-PGA synthesis [37]. Our results also showed that overexpressing *epsB* could reduce the expression of CcpA and CcpN while increasing the expression of NrgB, all of which improved the γ-PGA synthesis (Fig. 6).

### The fermentation production of *epsB* recombinant bacteria

**Fig. 7:**
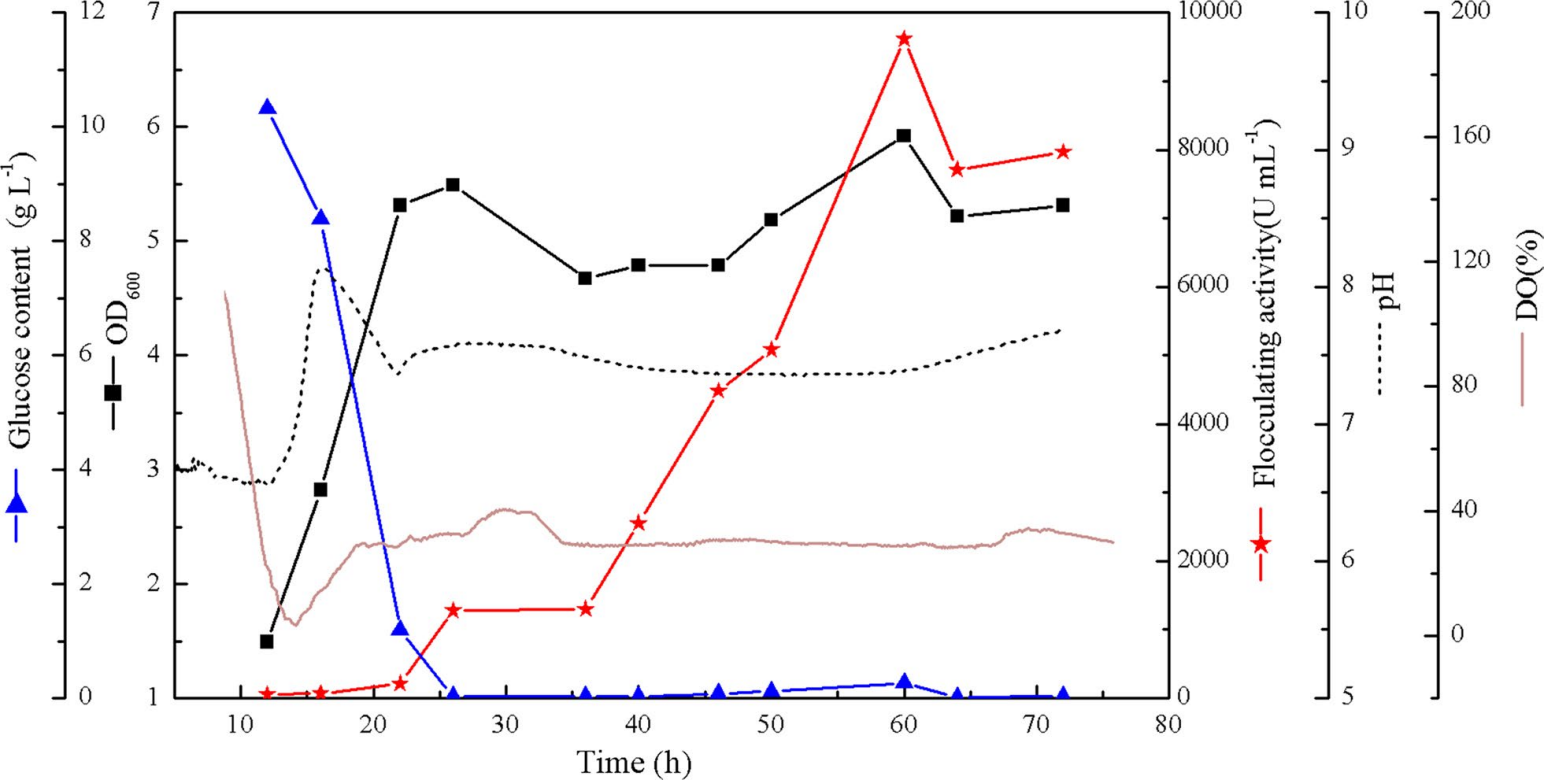
*Bacillus licheniformis*-phY300-*epsB* fermentation curve in 2 L fermenter

To evaluate the production of *B. licheniformis*-phY300-*epsB* in a more stable condition, batch fermentation was carried out in a 2 L fermenter. As shown in Fig. 7, the cells entered the stationary phase after 24 h of cultivation; at the same time, the glucose in the medium was used up, and then, the bacteria began to accumulate secondary metabolites. In addition, the flocculating activity of the fermentation broth began to increase rapidly until it peaked at 9612.75 U/mL after 60 h; this value was increased by 224% compared with the original strain under the same conditions and was 88.47% higher than the same strain cultured in the flask. Afterwards, the flocculating activity began to decline gradually. The final yield of the bioflocculant reached 10.26 g/L after alcohol precipitation and lyophilization; the yield was 36.62% higher than the original strain under the same conditions and 19.86% higher than the same strain cultured in the flask.

## Conclusions

In this study, we investigated the influence of overexpressing several key genes involved in polysaccharide biosynthesis on the flocculating activity and crude yield of polysaccharide bioflocculants in *B. licheniformis*. The results showed that overexpressing *epsA* enhanced the yield of the polysaccharide bioflocculants by 23.70%, and overexpressing the tandem gene *pgcA*-*gtaB1* enhanced the yield of the polysaccharide bioflocculants by 20.77% compared with that of the original strain, indicating that regulating the expression of *epsA* and *pgcA*-*gtaB1* promoted polysaccharide biosynthesis in *B. licheniformis*. Notably, overexpressing *epsB* from the *eps* gene cluster not only improved the flocculating activity of the bioflocculants by 117.92% to 5100.50 U/mL, compared with the wild-type strain, but also enhanced the crude yield of the bioflocculant by 13.98% to 8.56 g/L. Subsequently, focusing on the *epsB* recombinant strain *B. licheniformis*-pHY300-*epsB*, by analysing the composition of the bioflocculant produced by *B. licheniformis*-phY300-*epsB*, we found that overexpressing *epsB* increased the content of γ-PGA and decreased the total sugar content in the bioflocculant. By comparing the transcriptional levels of a few key genes involved in the biosynthesis of γ-PGA in *B. licheniformis*-phY300-*epsB* and the wild-type strain, we found that the transcriptional levels of *pgsA*, *pgsB*, *icd*, *rocA* and *nrgB* increased to different degrees, whereas the transcriptional levels of *ccpA* and *ccpN* decreased. Thus, we could determine that *epsB* played a key role in the biosynthesis of both the polysaccharides and γ-PGA. Moreover, overexpressing *epsB* markedly enhanced the γ-PGA synthesis during EPS fermentation and consequently improved the bioflocculant production. During batch fermentation in a 2 L fermenter, the flocculating activity of *B. licheniformis*-phY300-*epsB* reached 9612.75 U/mL, and the bioflocculant yield reached 10.26 g/L after 72 h, increasing 224% and 36.62%, respectively, compared with those of the original strain. These results reveal major potential for industrial production.

In conclusion, this study provides a new method to greatly increase the bioflocculant production in *B. licheniformis*, and it demonstrates the correlation between the biosynthesis of polysaccharides and γ-PGA during EPS fermentation by regulating the expression of EpsB. All of these results provide meaningful biological information for further research.

## Methods

### Bacterial strain and culture medium

The bacterium *B. licheniformis* CGMCC 2876 was isolated in our laboratory and deposited at the China General Microbiological Culture Collection Center (Beijing, China). The LB medium contained the following components (g/L): tryptone, 10; yeast extract, 5; and NaCl, 10, at pH 7.2. The preculture medium contained the following components (g/L): glucose, 10; urea, 0.5; MgSO_4_·7H_2_O, 0.2; KH_2_PO_4_, 0.1; K_2_HPO_4_, 0.1; NaCl, 0.1; and yeast extract, 0.5. The EPS medium consisted of the following components (g/L): glucose, 13.9; urea, 2.67; MgSO_4_, 0.048; KH_2_PO_4_, 5.6; K_2_HPO_4_, 1.4; NaCl, 2; and yeast extract, 0.6. For polysaccharide production, the initial pH of each medium was 7.2. The strain was first cultured at 200 rpm for 17 h in a 250 mL Erlenmeyer flask containing 50 mL of preculture medium at 37 °C. Subsequently, 4% of the seed culture (v/v) was inoculated at 200 rpm for 56 h in a 250 mL Erlenmeyer flask with 50 mL of EPS medium at 37 °C for polysaccharide production.

### Gene overexpression in *B. licheniformis*

The key genes were amplified using their corresponding primers (Table 1), and they were then cloned into the *E. coli*–*Bacillus* shuttle vector pHY300PLK-P*amyL*-TTamyL, which was constructed in our previous study. The recombinant expression vectors for genes *epsB*, *epsA*, *pgcA*, *gtaB1* and *crr* were designated as pHY300-*epsB*, pHY300-*epsA,* pHY300-*pgcA*, pHY300-*gtaB1*, and pHY300-*crr*, respectively. The tandem gene fragments *pgcA* and *gtaB1* were amplified using the primers *pgcA*-S and *pgcA*-A and *gtaB1*-S and *gtaB1*-A, respectively (Table 1), and the PCR products were spliced using a *pEASY*-Uni Seamless Cloning and Assembly Kit (TransGen Biotech, China). Using the assembled fragment as a template, the tandem gene *pgcA*-*gtaB1* was amplified using the *pgcA* forward primer and *gtaB1* reverse primer. Then, the tandem gene was cloned into pHY300PLK-P*amyL*-TTamyL by restriction digestion using *Kpn*I and *Spe*I. These recombinant expression vectors were separately transformed into *B. licheniformis* cells by electroporation, and the transformants were screened on tetracycline-resistant culture plates.

**Table 1:** Primers used in this study to amplify key genes

Name	Primer sequences (5′–3′)	Note
*crr*-F	GGGGTACCTTGCTGAAAAAATTATT	To amplify *crr*
*crr*-R	GGACTAGTTTACTTAACTTTAAGCTCCAT	
*pgcA*-F	GGGGTACCATGAAAGTAAAAAAAG	To amplify *pgcA*
*pgcA*-R	GGACTAGTTCAATTTGAAGTCGCTT	
*gtaB1*-F	GGGGTACCATGAAAGTAAAAAAAGC	To amplify *gtaB1*
*gtaB1*-R	CCACTAGTTCATTGCCATGCTCC	
*epsA*-F	GCCGGTACCATGAAAGAAAATATTG	To amplify *epsA*
*epsA*-R	GAAACTAGTCTAATAGCCAAGCGGC	
*epsB*-F	CCGGTACCTTGGCTATTAGAAAAAAAC	To amplify *epsB*
*epsB*-R	CCCACTAGTACATGGTTGCGTAATTAT	
*pgcA*-S	GAGGAAAATCGGTACATGAGCTGGAGAACGAG	To amplify tandem gene *pgcA*-*gtaB1*
*pgcA*-A	GACTGCTTTTTTTACTTTCATTCAATTTGAAGTCGCTTTTA	
*gtaB1*-S	TAAAAGCGACTTCAAATTGAATGAAAGTAAAAAAAGCAGTC	
*gtaB1*-A	CTTTTCTTCTCGAGATCATTGCCATGCTCCTT	

### Quantitative real-time PCR

The total RNA was extracted from *B. licheniformis* when the OD_600_ reached 3 by using the MiniBEST Universal RNA Extraction Kit (Takara, Japan).The RNA was quantified using a NanoDrop 2000 spectrophotometer (Thermo, USA). Using the High-Capacity cDNA Reverse Transcription Kit (Applied Biosystems, USA) for reverse transcription, the obtained cDNA was diluted to 100 ng/μL for further real-time PCR analyses performed on the stepOne Real-Time PCR System (Applied Biosystems, USA). The RT-PCR reaction was performed using Transtart Top Green qPCR Supermix Kit (TransGen Biotech, China) in a 20 μL mixture. Quantitative real-time PCR was performed in triplicate for each sample, and the relevant primers are listed in Table 2.

**Table 2:** Real-time PCR primers of key genes involved in polysaccharides synthesis

Name	Primer sequences (5′–3′)
q-*crr*-F	TCGGCATCCGTTCACTGT
q-*crr*-R	CCAAATCGCACGTTATCAAA
q-*pgcA*-F	AATGGAAGCTGCTAAAACGC
q-*pgcA*-R	AAAGGAAAGTTCAGGTGTCGG
q-*gtaB1*-F	GCAAAAGGAGCCCCTCGGTC
q-*gtaB1*-R	CGAGAAGGACGGCAAACGG
q-*epsA*-F	CAATACACGCGGACATTCCA
q-*epsA*-R	CTCTGATTCGCTTTCACTGCTC
q-*epsB*-F	ATCCTTTGAGAAGCCGTTTA
q-*epsB*-R	CCAAGTTTGAAGCCGAGAA

### Purification of the biopolymer and determination of the flocculating activity

After 56 h of fermentation, the culture broths were centrifuged at 9000 rpm for 15 min to remove the cells. Three volumes of ethanol were added to the supernatant to precipitate the crude products. Then, the crude products were dissolved using distilled water and lyophilized to obtain the purified products.

The flocculating activity (FA) was measured according to the method described by Kurane et al. [38]. First, 40 mL of 1% (wt/vol) kaolin and 2.5 mL of CaCl_2_ solution (10 g/L) were mixed thoroughly with 1 mL of the sample and then incubated for 5 min at room temperature. By measuring the decrease in turbidity (OD_550_) of the upper phase, the FA was calculated using the following equation: FA (U/mL) = (A − B)/A × 100 × D per mL, where A and B are the optical densities of the control and sample at 550 nm, respectively, and D is the dilution factor of the cell-free culture broth. Each sample was measured in triplicate.

### Analysis of the composition of the polysaccharide bioflocculant

The phenol–sulfuric acid method [39] was used to determine the total sugar content in the bioflocculant by measuring the optical densities of the samples at 490 nm. The protein content was measured using the Bradford method with the Bio-Rad Protein Assay Kit (Bio-Rad, USA) at OD_595_. The γ-PGA content in the polysaccharide bioflocculant was determined by HPLC with an Agilent 1200 HPLC system and an Agilent HC-C_18_ column (25 cm × 4.6 mm). The purified products were dissolved in 6 M HCl to hydrolyse the γ-PGA. The mixtures were maintained at 105 °C for 24 h and then neutralized and metered volumetrically. Afterwards, the samples were characterized using HPLC for qualitative and quantitative analysis. The operating conditions were as follows: 0.1 mol/L KH_2_PO_4_ (containing 5% methanol and with the pH adjusted to 2.5 using phosphoric acid) was used as the mobile phase at a flow rate of 1.0 mL/min; the injected volume was 20 μL, the column temperature was maintained at 30 °C, and the UV detection wave length was 210 nm. Pure sodium glutamate was used as the standard compound.

### Production of the polysaccharide bioflocculant from a batch culture in a 2 L fermenter

To cultivate larger volumes of the recombinant strain, 56 mL seed cultures of *B. licheniformis*-pHY300-*epsB* were cultivated in a seed medium in 250 mL shaker flasks for 17 h and then transferred into a 2 L fermenter system containing 1.4 L of the EPS medium. The pH, sterile air rate and the dissolved oxygen (DO) were maintained at 7.2, 2 vvm and 30%, respectively. The fermentation was carried out at 37 °C for 72 h to determine the final bioflocculant crude yield and flocculating activity.

## Additional file

**Additional file 1: Table S1.** Provides the related primers used in the Fig. 5.

## Abbreviations

EPS: extracellular polysaccharides
γ-PGA: poly-gamma-glutamic acid
TCA: tricarboxylic acid cycle
UTP: uridine triphosphate
UDP: uridine diphosphate
HPLC: high-performance liquid chromatography
