# Supplementary material for: Increasing the bioflocculant production and identifying the effect of overexpressing epsB on the synthesis of polysaccharide and γ-PGA in Bacillus licheniformis
Source: Microb Cell Fact. 2017 Sep 26;16:163. doi: 10.1186/s12934-017-0775-9 (PMC5615475; doi:10.1186/s12934-017-0775-9)
Supplement: Supplementary file 1 — Additional file 1: Table S1. Provides the related primers used in the Fig. 5. [file 12934_2017_775_MOESM1_ESM.docx]

**Table S1** **Real-time PCR primers of key genes involved in γ-PGA synthesis**

| Name | Primer sequences(5’ to 3’) |
| --- | --- |
| q*-rocA-*F | GTAAATAACGAATGGCTGGGTC |
| q*-rocA-*R | CCTTGGATACCGTGCCTACA |
| q*-icd-*F | CGGTCATCCCGTTTATTGA |
| q*-icd-*R | CGGCGTAGACTTCTTTCCA |
| q*-pgsA*-F | TGAGGACTTGGAGCTTGTCG |
| q*-pgsA*-R | TTGGCTGTAAAGTTCGTGTAG |
| q*-pgsB*-F | TGATGGGACCGACTTTGGAT |
| q*-pgsB*-R | TGTCTGCGACGATGACTTTT |
| q*-pgsC*-F | GTGATGCCGTTTGAGATTGC |
| q*-pgsC*-R | CATAATGACGAATGTTGCTCC |
| q*-ccpA*-F | CAAACCTCAAAACCTACAG |
| q*-ccpA*-R | TCACGAATAAGAGACAGCA |
| q*-ccpN*-F | AATGTCTCCGTTTATGATGC |
| q*-ccpN*-R | CTGTTTGCCGATGCTGG |
